# Epigenetic Regulation of miR-92a and TET2 and Their Association in Non-Hodgkin Lymphoma

**DOI:** 10.3389/fgene.2021.768913

**Published:** 2021-11-26

**Authors:** Esther K. Elliott, Lloyd N. Hopkins, Robert Hensen, Heidi G. Sutherland, Larisa M. Haupt, Lyn R. Griffiths

**Affiliations:** ^1^ Centre for Genomics and Personalised Health, Genomics Research Centre, School of Biomedical Sciences, Queensland University of Technology (QUT), Kelvin Grove, QLD, Australia; ^2^ Icon Cancer Centre, Brisbane, QLD, Australia

**Keywords:** miR-17∼92, Burkitt lymphoma, mantle cell lymphoma, diffuse large B-cell lymphoma, micro-RNA (miRNA/miR), DNA methylation, epigenetic regulation

## Abstract

MicroRNAs (miRNAs) are well known for their ability to regulate the expression of specific target genes through degradation or inhibition of translation of the target mRNA. In various cancers, miRNAs regulate gene expression by altering the epigenetic status of candidate genes that are implicated in various difficult to treat haematological malignancies such as non-Hodgkin lymphoma by acting as either oncogenes or tumour suppressor genes. Cellular and circulating miRNA biomarkers could also be directly utilised as disease markers for diagnosis and monitoring of non-Hodgkin lymphoma (NHL); however, the role of DNA methylation in miRNA expression regulation in NHL requires further scientific inquiry. In this study, we investigated the methylation levels of CpGs in CpG islands spanning the promoter regions of the miR-17–92 cluster host gene and the TET2 gene and correlated them with the expression levels of *TET2* mRNA and miR-92a-3p and miR-92a-5p mature miRNAs in NHL cell lines, tumour samples, and the whole blood gDNA of an NHL case control cohort. Increased expression of both miR-92a-3p and miR-92a-5p and aberrant expression of TET2 was observed in NHL cell lines and tumour tissues, as well as disparate levels of dysfunctional promoter CGI methylation. Both miR-92a and TET2 may play a concerted role in NHL malignancy and disease pathogenesis.

## Introduction

Non-Hodgkin Lymphoma (NHL) is a class of cancer that originates in the lymphatic system, caused by an over-proliferation of malignant B-cells. NHL is one of the most common cancers in the United States, and lymphoma is the fifth most common cancer in Australasia (Abba et al., 2016), therefore posing a significant health burden (Calin et al., 2002; Cancer Council, 2020). The five-year survival rate is an estimated 71% (Allemani et al., 2018); however, both outcomes and pathogenesis vary greatly between NHL subtypes. Of the more than 40 subtypes of NHL, the two most common subtypes consist of the indolent follicular lymphoma (FL), and the more aggressive diffuse large B-cell lymphoma (DLBCL). Aggressive subtypes account for approximately 60% of cases, and a significant number of NHL patients suffer relapse on various treatments, with refractory NHL having a much poorer prognosis despite access to extensive chemotherapy and immunotherapy regimes (Michot et al., 2018; Sarkozy and Sehn, 2018; Ayers et al., 2020). Overall, this makes NHL an intractable disease to manage and treat, highlighting the need for reliable and subtype-specific diagnostic and prognostic biomarkers.

**GRAPHICAL ABSTRACT:**
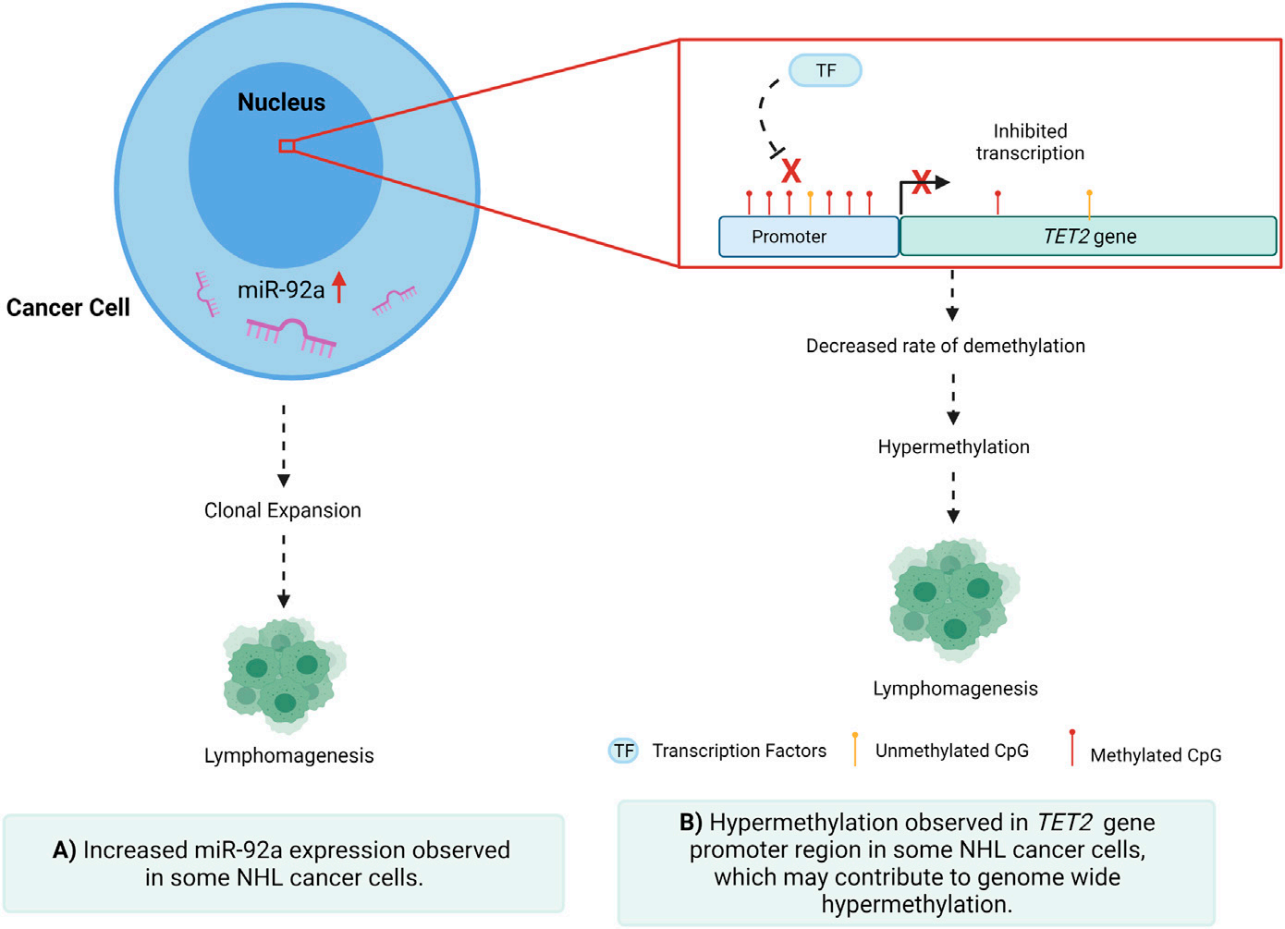


Understanding the cell of origin also plays a significant role in understanding NHL classification, progression, and prognosis (Alizadeh et al., 2000). B-cell lymphomas account for approximately 80% of NHL cases (Shankland et al., 2012), with DLBCL making up 25% of NHL diagnoses (Teras et al., 20162016; Siegel et al., 20192019). In 2000, gene expression profiling further defined DLBCL into two molecular subtypes; germinal centre B-cell like (GCB) and activated B-cell like (ABC) (Alizadeh et al., 2000). Also known as non-GCB, ABC demonstrated a poorer response to standard immunochemotherapy when compared to DLBCL cases with no specific subtype. This has been in part attributed to two oncogenic mechanisms seen in this subtype, encompassing the prevention of apoptosis and blockage of terminal differentiation (Blenk et al., 2007). These new subtypes of DLBCL, termed high-grade B-cell lymphoma, double-hit lymphoma, or triple hit lymphoma are associated with MYC and BCL2 and/or BCL6 rearrangements (Blenk et al., 2007) which are believed to contribute to their more aggressive oncogenesis.

Dysregulation of miRNAs is a hallmark of both cancer initiation and metastasis, through the regulation of gene expression via post-transcriptional repression and mRNA degradation (Di Lisio et al., 2012; Martin-Guerrero et al., 2015; Bradshaw et al., 2016; Getaneh et al., 2019). It is well established that miRNAs can act as both oncogenes and tumour suppressors in several cancers (Esquela-Kerscher and Slack, 2006; Di Lisio et al., 2012; Caramuta et al., 2013; Martin-Guerrero et al., 2015; Bradshaw et al., 2016; Getaneh et al., 2019), including NHL, via targeted repression of regulatory factors involved in processes such as cellular proliferation and migration (Xiao et al., 2008; Lawrie et al., 2009; Culpin, 2010). Previous studies have identified numerous miRNAs as having oncogenic potential in NHL (Amodio et al., 2015; Gado et al., 2019), as well as several being identified to be differentially expressed in tissues and fluids of individuals with these diseases. It has been previously established that miR-92a-3p has oncogenic potential, implicated in several cancers, including NHL, proposing that dysregulation of this miRNA may play a role in disease development (Culpin, 2010; Amodio et al., 2015; Gado et al., 2019). In both leukemia and DLBCL, miR-92 was seen to be downregulated, raising the question of whether this same downregulation is seen in indolent NHL subtypes such as FL (Amodio et al., 2015; Gado et al., 2019). The oncogenic miR-17–92 cluster, which includes miR-92a-3p, is a known driver of Burkitt lymphoma (BL), wherein miR-17 is associated with a poor prognosis and decreased overall survival rate (Robaina et al., 2016). miR-17–92 regulates multiple cellular pathways within NHL subtypes that favour malignant transformation, cellular proliferation, and cell survival (Dal Bo et al., 2015). In GCB DLBCL, the mir-17–92 microRNA cluster has been shown to be over-expressed when compared to ABC DLBCL (Lenz et al., 2008). Comparatively, the miR-92a-5p mature miRNA is not well described in NHL. With these relationships in mind, oncogenic or tumour suppressive miRNAs may be feasible targets for therapeutic agents, with the possibility of slowing or even halting malignancy (Wang and Wu, 2009), (Arif et al., 2020; Otoukesh et al., 2020). As recently reviewed in an earlier publication by our group, miRNAs can be epigenetically regulated, and even self-mediate these epigenetic modulations in various cancers (Arif et al., 2020). Aberrant DNA methylation in cytosines and adenines of mature miRNAs can lead to downregulation and suppression of the specific miRNA and, in NHL, a greater likelihood of lymphomagenesis (Calin and Croce, 2006; Esquela-Kerscher and Slack, 2006; Abba et al., 2017; Arif et al., 2020). These relationships support the possibility of miRNAs as being both functionally relevant in NHL and possible therapeutic targets.

The TET2 gene primarily functions as a tumour suppressor gene, immune regulator, and driver of DNA repair, as well as playing a key role in DNA demethylation (Fang et al., 2012). The TET2 protein catalyses the conversion of 5-methylcytosine (5mC) to 5-hydroxymethylcytosine (5-hmC), playing an integral role in transcriptional regulation. It is plausible that a hypermethylated genome may be due to a combination of factors that include high expression of DNA methyltransferases (DNMTs) or low expression of TET proteins (Fang et al., 2012; Asmar et al., 2013). TET2 is also involved in the recruitment of O-GlcNAc transferase OGT to CpG-rich sites to promote histone H2B GlcNAcylation (Asmar et al., 2013; Wang et al., 2018). TET2 is highly expressed in stem and progenitor cells, and in T-helper cells, whereas loss of *TET2* gene function in bone marrow cells leads to increased immature B-cells resulting in lymphomagenesis (Fang et al., 2012; Asmar et al., 2013; Chiba, 2017). In NHL, TET2 expression is often reduced and regulated by methylation (Chiba, 2017). Various pathways are hypothesised to be regulated by miR-92a-*TET2* including; JAK-STAT signalling, human pluripotent stem cells (hPSCs) differentiation, balanced B-cell terminal differentiation, and oxidative demethylation (miRTarBase, 2021; mimirna, 2021). To date, the prognostic significance of TET2 dysregulation in NHL is still not well characterised and further understanding of epigenetic drivers of *TET2* regulation in lymphoma requires additional investigation.

## Materials and Methods

### An Australian NHL and Healthy Volunteer Cohort

A case-control cohort was previously recruited (Bradshaw et al., 2015), and a sub-cohort consisting of the peripheral blood gDNA of 80 retrospective case samples of Caucasian origin with Australian/British/European grandparents and with no family history of a haematological malignancy was assembled for this study. Details on the clinical diagnosis of individuals in the NHL patient cohort are listed in Table 1. The healthy control cohort consisted of 80 healthy individuals of similar age who had not been diagnosed with and cancer, nor did they have a family history of haematological malignancy. Cases were matched according to age (within ±5 years), sex, and ethnicity with the healthy control samples. For the 80 cases and controls, 26 (32.5%) were male and 54 (67.5%) were female. The mean age of the NHL diagnosed individuals was 66.56 years at collection in 2013, with a standard deviation of 12.40 years; the mean age of the control cohort was 64.54 years at collection in 2013, with a standard deviation of 11.61 years.

**TABLE 1:** GLP NHL case cohort with subtypes and number of cases for each subtype listed.

NHL Subtype	Number of cases
FL	23
DLBCL	14
Chronic lymphocytic leukemia (CLL)	5
Lymphoblastic lymphoma (LBL)	5
MCL	3
Mucosa-associated lymphoid tumour (MALT)	2
BL	2
Other B-Cell Lymphoma	26
Total	80

Snap-frozen lymph node tumour biopsies were obtained from BioOptions (California United States), with tumour tissue from 11 NHL cases (4 female and 7 female) with a confirmed diagnosis of NHL (5 DLBCL and 6 FL) (Table 2). A cohort of healthy volunteers was used for controls to compare with cell lines and tumours, using PB-derived leukocytes from 6 individuals aged between 23 and 38 years, with a mean age of 30 years and a standard deviation of 5.65, who had not been diagnosed with cancer nor did they have a family history of haematological malignancy. Of the 6 healthy controls, 3 (50%) were female.

**TABLE 2:** Pathology of NHL tumour samples, including immunohistochemistry and comments from pathologist.

GLP ID	Diagnosis	Gender	Age	Anatomy	Immunohistochemistry	Pathologist comments
DLBCL03	DLBCL	M	71	Bowel	CD45^+^, CD20^+^, CD30^+^, CD15^−^	Multilobulated Reed-Sternberg like cells, extension into perinodal soft tissue
DLBCL04	DLBCL	F	57	Groin	CD10^+^, CD20^+^, CD5^−^, CD3^+^, BCL-2+, BCL-6+, Ki-67+, CD1^+^, CD30^−^	Consistent with transformed lymphoma
DLBCL05	FL; DLBCL	F	52	Bowel	CD20^+^, BCL2+, CD10^+^, BCL6+, Ki67+	Not tested
DLBCL06	DLBCL	F	68	Axillary	CD20^+^, BCL6+, BCL2+, MUM1+, CD3^−^, CD10^−^, CD138-	Non-GCB origin
DLBCL09	DLBCL	F	50	Submandibular	CD20^+^, BCL2+, CD10^−^, CD3^−^, Kappa, Ki67+	t(6:22)
DLBCL10	DLBCL	M	87	Supraclavicular	CD20^+^, CD30^+^, Ki67+, BLC2 +, BCL1-, CD3^−^, CD5^−^, CD10^−^, CD23^−^	Negative for cytogenetics
FL01	FL	M	60	Testis	CD20^+^, CD3^+^ T-cells, BCL2-	Not tested
FL03	FL	M	62	Groin	CD20^+^, CD19^+^ and FMC7, CD10, CD5^−^	Not tested
FL08	FL	F	47	Groin	CD3^−^, CD20^+^, BCL2+, Kappa+, Lambda-	Not tested
FL09	FL	F	59	Groin	Ki67+, CD10^+^, CD20^+^, CD19^+^	Not tested
FL10	FL	F	63	Axillary	CD10^+^, BCL2+, CD23^−^, CD3 T-cell, CD5^+^ T-cell, ki67+	Not tested

### Cell Culture of B-NHL Cell Lines

Studies were conducted using four commercial immortalised cell lines: Raji (BL), Toledo (DLBCL), SUDLH4 (DLBCL), and Mino (Mantle cell lymphoma (MCL)). Vials were stored in liquid nitrogen and rapidly thawed and seeded in a T-75 culture flask in 25 ml RPMI-1640, supplemented with 10% FBS and 1% Penicillin-Streptomycin antibiotics. The cells were incubated at 37°C in a humidified 5% CO_2_ incubator. Cell count and viability were assessed using Trypan Blue exclusion method in a 1:1 ratio of cell suspension to dye and measured on a TC10™ automated cell counter. Cells were split into a 1:2 ratio, with fresh media was added every second day until >80% confluence and 90% viability were achieved.

### Computational Analysis and Target Identification

Computational analysis of miRNA and target gene prediction was performed using miRbase (miRBase, 2021), miRTarBase (miRTarBase, 2021), and Target Scan (Target Scan, 2021), identifying miR-92a-3p and 5p as candidates. DNA methylation analysis in the miR-17–92 cluster host-gene (*MIR17HG*) promoter region and the miR-92a to *TET2* promoter was performed using MethHC 2.0 software.

### Nucleic Acid Extraction, Bisulfite Conversion, and Reverse Transcription

gDNA was previously extracted from whole blood, collected into EDTA tubes, using an in-house salting-out method as evaluated by Chacon et al. (Chacon-Cortes et al., 2012). Both NHL cell line, control peripheral blood mononuclear cell (PBMC), and NHL tumour sample gDNA was extracted using the Wizard^®^ SV Genomic DNA Purification System (Promega). DNA quality and quantity were quantified using a Nanodrop spectrophotometer, determined by A260/A280 ratio (ND1000 V3.8.1, ThermoFisher Scientific).

Bisulfite conversion of 500 ng of NHL B-cell line and control PBMC DNA and 750 ng of DNA from each patient/control DNA sample and NHL tumour sample was performed using the EZ DNA Methylation™ Kit (Zymo Research) according to the manufacturer’s protocol, with a modification to the elution volume, from 10 to 40 µL.

Total RNA was extracted using TRIzol^®^ Reagent (ThermoFisher) and the Direct-zol RNA MiniPrep Kit (Zymo Research) from cell lines, control PBMCs, and NHL tumour samples. Total RNA, including miRNA, was reverse transcribed using the MiScript II RT Kit (Qiagen) for all samples.

### Quantitative PCR of miRNA and mRNA Expression

Analyses were performed by real-time quantitative PCR (Q-PCR) to measure miR-92a mature miRNAs (MiScript SYBR^®^ Green PCR, Qiagen) and mRNA transcripts (SYBR^®^ Green PCR master mix, Promega). Customised forward and reverse primers (Integrated DNA Technologies, Inc) were designed for the miRNAs and *TET2* mRNA transcripts of interest (Table 3). All assays were performed on the QuantStudio™ 7 Flex Real-Time PCR System (ThermoFisher) running the QuantStudio™ Software (v1.7.1) (ThermoFisher). miRNA assays were performed under the following conditions: 95°C for 2 min s (x1 cycle), 95°C for 10 s, 56°C for 60 s (x40 cycles), melt curve analysis 60–95°C. *TET2* expression was measured under the following conditions: 50°C for 2 min (x1 cycle), 95°C for 3 min (x1 cycle), 95°C for 3 s, 60°C for 30 s (x50 cycles), melt curve analysis 60–95°C. Endogenous controls were miR103 for the miRNA assays and 18 S ribosomal RNA for the *TET2* assays. Specific amplification of targets was confirmed via melt curve analysis and gene expression was calculated using the relative quantification method (ΔΔCt).

**TABLE 3:** Gene expression forward and reverse primer sequences with transcript accession numbers. miRNA expression assays utilised miScript universal reverse primer (Qiagen).

Gene		Primer Sequence (5’—3’)	Product size	Accession
TET2	F	TGG​CAA​ACA​TTC​AGC​AGC​AC	153 bp	NM_001127208.2
	R	AGT​TGA​ATT​CAG​CAG​CTC​AGT		NM_017628.4
92a-5p	F	GAGGTTGGGATCGGTTG	∼87 bp	MIMAT0004507
92a-3p	F	CGCAGTATTGCACTTGC	∼87 bp	MIMAT0000092

### DNA Methylation Studies

**FIGURE 1:**
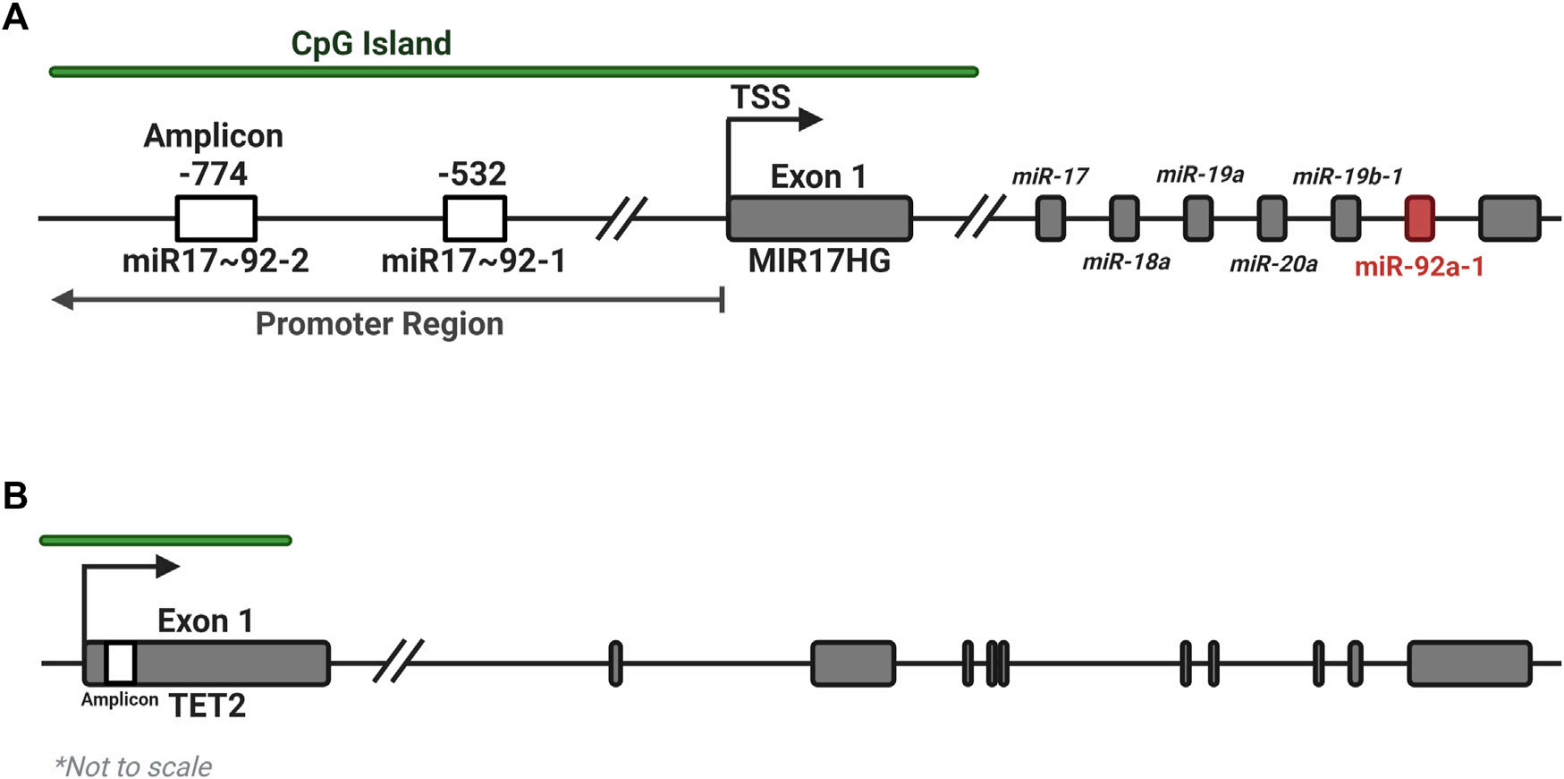
Location of amplicons for the MIR17HG and TET2 methylation assays. Two regions of four CpGs each were identified within the promoter CGI of the MIR17HG gene **(A)**, and one region in the first exon of the TET2 gene **(B)**, each containing several regulatory motifs and transcription factor binding sites. Created with BioRender.com.

The UCSC genome browser was used to identify CGI regions in the *MIR17HG* and *TET2* promoter regions (Figure 1). Pyromark Assay Design 2.0 software (Qiagen) was used to generate amplification and pyrosequencing primers to target regions in the CGI containing numerous CpGs (Table 4). Primers were designed to incorporate biotin on the reverse primer and an additional sequencing primer for the pyrosequencing, with one set targeting 7 CpGs in Exon 1 of *TET2* and two regions of four CpGs were targeted within the promoter CGI of the *MIR17HG* gene, both regions that containing several transcription factor binding sites. Pyrosequencing was performed on a PyroMark Q48 Autoprep system (Qiagen) as per the manufacturer’s instructions using PyroMark Q96 Gold reagents (Qiagen). Pyrosequencing output analysis was performed using the Qseq software (BioMolecular Systems, V2.4.3).

**TABLE 4:** PCR and Pyrosequencing primers for TET2 and MIR17HG regulatory regions.

Gene	Primer Sequence (5’—3′)
TET2 prom CGI Forward primer:	GGAGTAGGGGTTAGGGTT
TET2 prom CGI Reverse primer:	bio-ACTCTACTTCTTCTCCCAAAAAT
TET2 prom CGI sequencing primer:	TAGGGGTTAGGGTTG
miR-17–92 R1 Forward primer:	GGT​TGG​TTT​GGA​GTA​GGT​TTT​TAA​TT
miR-17–92 R1 Reverse primer:	bio-CTTCCCCAAACTTCCTAAAAACCCTACTCT
miR-17–92 R1 Sequencing primer:	GGT​AGG​TAA​AGT​AAT​AAA​TTG​TGA​T
miR-17–92 R2 Forward primer:	GAG​GGG​AGG​TTT​AGG​TAT​TG
miR-17–92 R2 Reverse primer:	bio-AAACCCAAAAATAAATACATTACACCC
miR-17–92 R2 Sequencing primer:	TGT​AGT​TGT​GAA​ATT​TTT​GT

### Statistical Analysis

Statistical analysis of both expression and methylation data was performed in GraphPad Prism, with a Kruskal–Wallis (KW) test of significance and a post-hoc Dunn test for comparisons of individual groups.

## Results

### Differential miR-92a and TET2 Expression and Differential Upstream Promoter CGI Methylation Were Observed Between NHL Cell Lines and Healthy Control PBMCs

**FIGURE 2:**
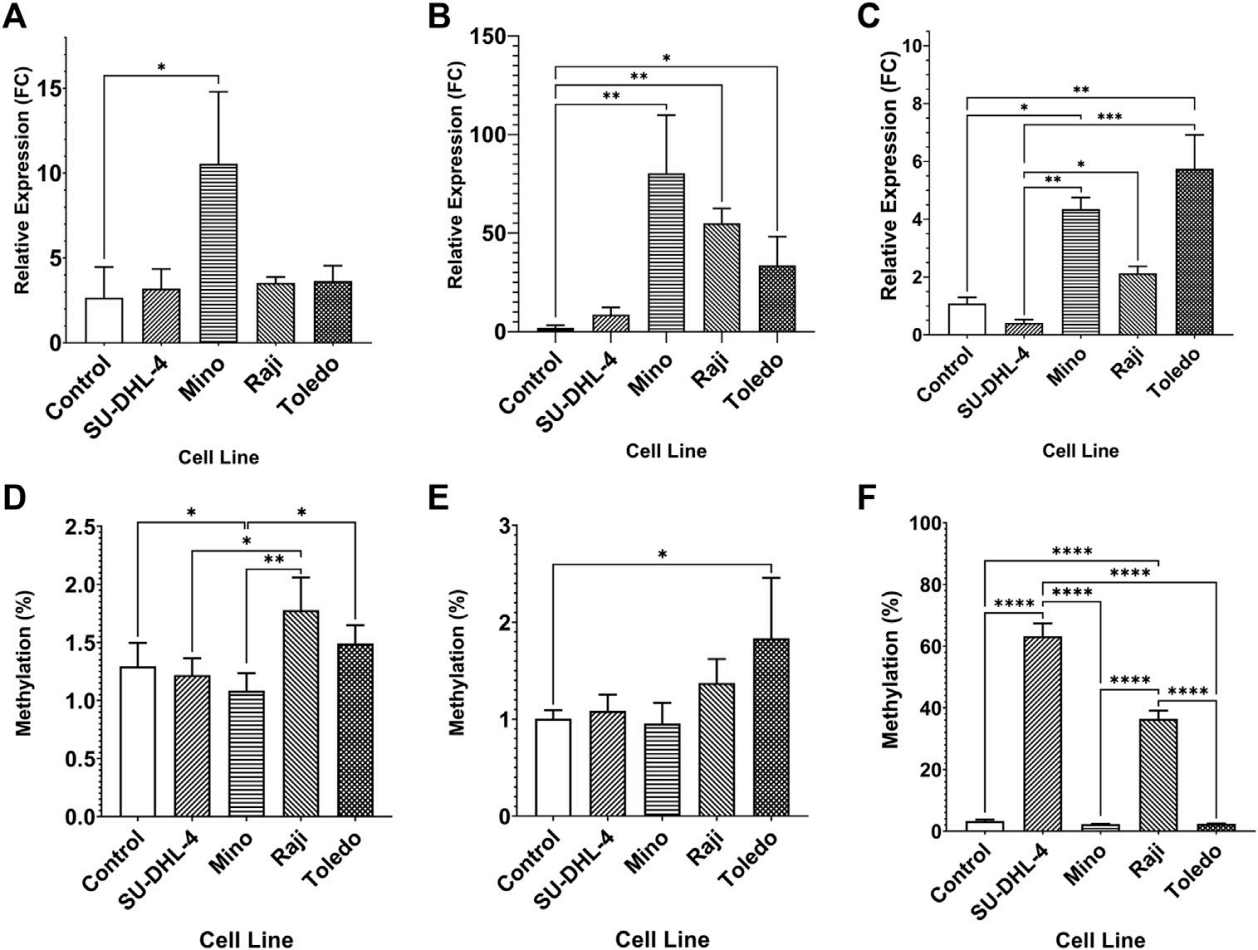
Expression miRNA 92a-3p, miRNA 92a-3p, and TET2 mRNA, and promoter DNA methylation of CpGs in two regions of the promoter CGI of the miR-17∼92 cluster and one region in the promoter CGI of the TET2 gene in NHL cell lines and in healthy control PBMCs. The expression of miR-92a-3p **(A)**, miR-92a-5p **(B)** and TET2 mRNA **(C)** was assayed by RT-qPCR in commercially available NHL cell lines SU-DHL-4, Mino, Raji, and Toledo, and in healthy control PBMCs. Bisulfite pyrosequencing was performed on two regions in the promoter CGIs of the miR-17–92 cluster and in one region of the TET2 gene in the same cells and controls. Mean methylation levels of 4 CpGs in the two regions, 532 bp **(D)** and bp **(E)** upstream of the *MIR17HG* TSS were assayed, and mean methylation levels of 6 CpGs in the first exon of TET2 were also assayed **(F)**. Bars denote mean and error bars denote SEM. Statistical significance calculated by KW test of significance and a post-hoc Dunn test for comparison of cell lines to controls, with significance denoted as: **** *p* < 0.0001, *** *p* < 0.001, ** *p* < 0.01, * *p* < 0.05.

The expression of miR-92a-3p and miR-92a-5p mature miRNAs and *TET2* mRNA were assayed by RT-qPCR, and upstream promoter CGI methylation for each gene was assayed by bisulfite pyrosequencing, in 4 biological replicates of NHL cell lines SU-DHL-4, Mino, Raji, and Toledo, and in PBMCs of 6 healthy controls (Figure 2). miR-92a-3p expression was increased in Mino cells compared to controls (Figure 2A, *p* = 0.0145) and miR-92a-5p expression increased in Mino (*p* = 0.0013), Raji (*p* = 0.0016), and Toledo (*p* = 0.0200) cells compared to controls (Figure 2B). Differential methylation was observed in the *MIR17HG* upstream region, with Mino cells showing decreased methylation in region 1 when compared to controls (Figure 2D, *p* = 0.0460). Differential methylation was also observed between SU-DHL-4 and Raji cells (*p* = 0.0237). and between Mino cells and Raji (*p* = 0.0029) and Toledo (*p* = 0.0278). Toledo (*p* = 0.0329) cells showed increased methylation in region 2 when compared to controls (Figure 2E).

*TET2* was differentially expressed in Mino (*p* = 0.0153) and Toledo (*p* = 0.0045) cells compared to controls (Figure 2C). Differential expression was also observed between SU-DHL-4 cells and Mino (*p* = 0.0016), Raji (*p* = 0.0385), and Toledo (*p* = 0.0004) cells. Upstream promoter hypermethylation was observed in SU-DHL-4 and Raji cell lines when compared to controls. (Figure 2F, *p* < 0.0001). SU-DHL-4 cells were also significantly different to Mino and Toledo cells (*p* < 0.0001), and Raji cells were significantly different to Mino and Toledo cells (*p* < 0.0001).

### Differential miR-92a and TET2 Expression and Differential Upstream Promoter CGI Methylation Were Observed Between Healthy Control PBMCs and NHL Tumours

**FIGURE 3:**
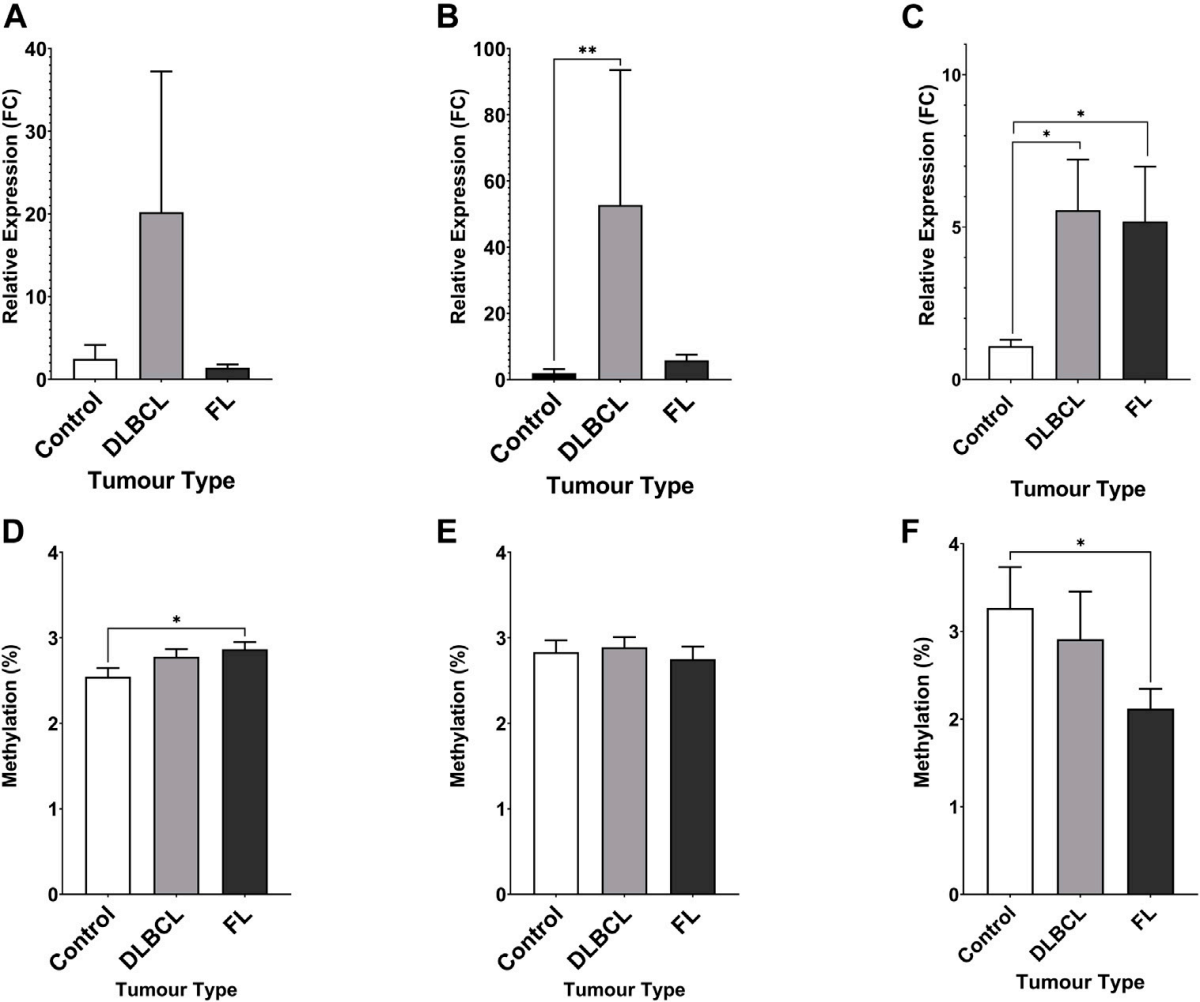
Expression miRNA 92a-3p, miRNA 92a-3p, and TET2 mRNA, and promoter DNA methylation of CpGs in two regions of the promoter CGI of the miR-17∼92 cluster and one region in the promoter CGI of the TET2 gene in NHL tumours compared to healthy control PBMCs. The expression of miR-92a-3p **(A)**, miR-92a-5p **(B)** and TET2 mRNA **(C)** was assayed in by RT-qPCR in malignant lymphatic tissue of NHL patients and in healthy control PBMCs. Bisulfite pyrosequencing was performed on two regions in the promoter CGIs of the miR-17–92 cluster and in one region of the TET2 gene. Mean methylation levels of 4 CpGs in the two regions, 532 bp **(D)** and 774 bp **(E)** upstream of the MIR17HG TSS were assayed, and mean methylation levels of 6 CpGs in the first exon of TET2 were also assayed **(F)**. Bars denote mean and error bars denote SEM. Statistical significance calculated by KW test of significance and a post-hoc Dunn test for comparison of cell lines to controls, with significance denoted as: **** *p* < 0.0001, *** *p* < 0.001, ** *p* < 0.01, * *p* < 0.05.

The expression of miR-92a-3p and miR-92a-5p mature miRNAs and *TET2* mRNA were assayed in NHL patient tumour biopsies and compared to healthy control PBMCs by RT-qPCR (Figure 3). Promoter methylation for each gene was assayed by bisulfite pyrosequencing in the same cohort. miR-92a-5p expression was increased in DLBCL tumours compared to controls (Figure 3B, *p* = 0.0017), and differential methylation was observed between controls and FL tumours in region 1 of the *MIR17HG* upstream promoter CGI (Figure 3D, *p* = 0.0203).

Increased *TET2* mRNA expression was observed in both DLBCL (*p* = 0.0164) and FL (*p* = 0.0240) patient tumour biopsies compared to controls (Figure 3C), and decreased methylation was observed in FL tumours compared to controls (Figure 3F, *p* = 0.0300).

### Aberrant Upstream Promoter CGI Methylation of MIR17HG and TET2 Identified in NHL Patient and Healthy Control Whole Blood gDNA

**FIGURE 4:**
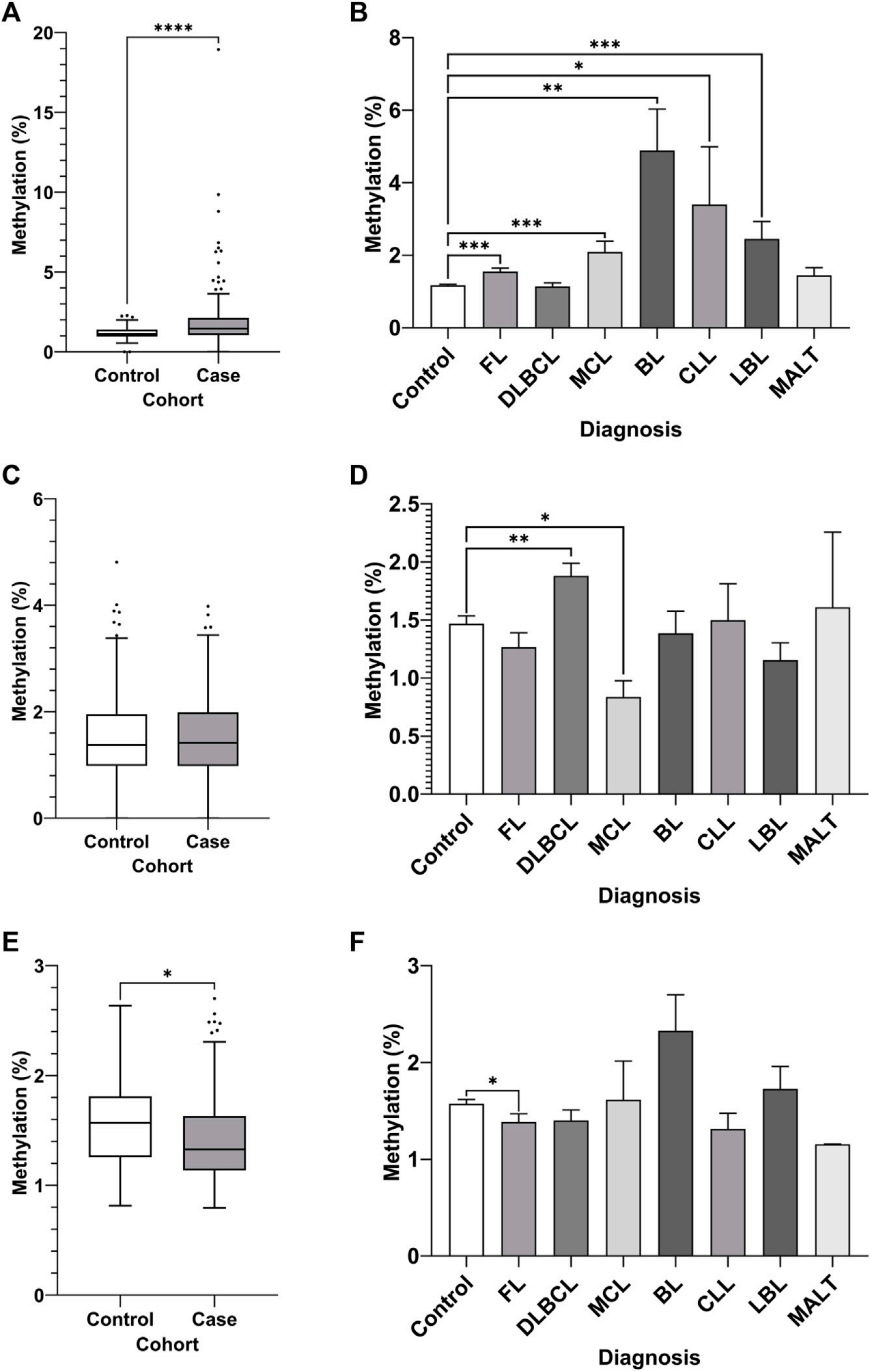
Promoter DNA methylation of CpGs in two regions of the promoter CGI of the miR-17∼92 cluster and one region in the promoter CGI of the TET2 gene in whole blood gDNA of a retrospective NHL case-control cohort. Bisulfite pyrosequencing was performed on two regions in the promoter CGIs of the miR-17–92 cluster and in one region of the TET2 gene. Mean methylation levels of 4 CpGs in the two regions, 532 bp **(A–B)** and 774 bp **(C–D)** upstream of the MIR17HG TSS were assayed, and mean methylation levels of 6 CpGs in the first exon of TET2 were also assayed **(E–F)**. Patients were categorised by NHL subtype, with mean methylation levels compared between subtype and controls. Individuals without a specific subtype were not included in comparison between diagnosis. Bars denote mean and error bars denote SEM. Statistical significance calculated by KW test of significance and a post-hoc Dunn test for comparison of cell lines to controls, with significance denoted as: **** *p* < 0.0001, *** *p* < 0.001, ** *p* < 0.01, * *p* < 0.05.

DNA methylation of CpGs in each region was assayed by bisulfite pyrosequencing of whole blood gDNA from an NHL case-control cohort, comprised of 80 cases with age and sex-matched controls (Figure 4). Increased DNA methylation of region 1 of the *MIR17HG* promoter CGI was observed in the NHL cohort compared to the controls (Figure 4A, *p* < 0.0001). Furthermore, differential DNA methylation was observed between healthy controls and NHL patient subtypes, with increased levels of DNA methylation observed in the FL (*p* = 0.0004), MCL (*p* = 0.0046), BL (*p* = 0.0062), and LBL (*p* = 0.0004) patient groups (Figure 4B). No differential methylation was observed between the cases and controls in region 2 of the *MIR17HG* upstream promoter CGI (Figure 4C); however, differential methylation was observed between controls and the DLBCL (*p* = 0.0041) and MCL (*p* = 0.0398) subtypes when the specific diagnosis was considered (Figure 4D). Decreased *TET2* promoter CGI methylation was observed in NHL cases compared to controls (Figure 4E, *p* = 0.0159), and between healthy controls and FL subtype (Figure 4F, *p* = 0.0210).

## Discussion

In the last decade, both the role of miRNAs in malignancy and their viability as biomarkers for malignancy has been a rapidly expanding area of research. The mechanisms behind miRNA expression are not fully understood; however, aberrant methylation of promoter regions of miRNA genes has been previously implicated in aberrant miRNA expression in several cancers (Weber et al., 2007; Suzuki et al., 2010; Suzuki et al., 2012; Harada et al., 2015), including lymphoma (Stumpel et al., 2011; Yim et al., 2012). The expression profiles of miRNAs have been previously presented as novel biomarker panels for NHL (Khare et al., 2017; Solé et al., 2017), but the role of DNA methylation in miRNA expression regulation in NHL is not well explored. In this study, the methylation levels of CpGs in CGIs spanning the promoter regions of the miR-17–92 cluster host gene and the *TET2* gene were quantified by bisulfite pyrosequencing and correlated with the expression levels of *TET2* mRNA and mature miR-92a-3p and miR-92a-5p miRNAs. Gene expression was assayed in NHL B-cell lines, NHL tumour samples, and healthy PBMCs. Methylation of promoter CGIs was measured in the DNA of several NHL B-cell lines, in NHL tumour samples, in healthy PBMCs, and in whole blood gDNA of a retrospective NHL case-control cohort.

Increased expression of both the −3p and −5p mature miRNAs was observed in Mino cells when compared to controls, and additionally, upregulation of −5p was observed in Raji and Toledo cells lines. Previous studies have identified miR-92a overexpression in BL, MCL, and DLBCL cell lines, and our study replicates these findings (He et al., 2005; Ji et al., 2011). Higher expression of miR-92a has also been previously identified in MCL tumours (Husby et al., 2013; Roisman et al., 2016), reflecting the increased expression identified in Mino cells in this study. Increased expression of miR-92a-3p was also observed in our DLBCL tumour samples when compared to controls. Increased expression of miR-92a has previously been found to cause lymphatic malignancy in mice (Xiao et al., 2008), with downregulation of miR-92a implicated in the inhibition of tumour growth in an MCL mouse model (Rao et al., 2012). Overexpression of miR-92a and has been previously implicated in the reduction of overall survival and event-free survival in NHL patients (Yan et al., 2019), reinforcing the relevance of our findings.

Differential methylation was observed in the upstream promoter CGI of *MIR17HG* between cell lines, and between cell lines and healthy controls; however, this difference may not be biologically significant as the difference between mean methylation is within the detection limit margin of error (Gruber et al., 2002; Tost and Gut, 2007). Therefore, it seems unlikely that methylation of CpGs in these specific regions in the *MIR17HG* promoter region contribute to the differential miR-92a expression between NHL cell lines. A similar trend was identified in the NHL tumour samples; however, again the difference between mean methylation is within the detection limit margin of error. Aberrant methylation of the *MIR17HG* promoter has been previously correlated with dysfunctional expression of miRNAs in the miR-17–92 cluster in lung biopsies of individuals diagnosed with pulmonary fibrosis (Dakhlallah et al., 2013), and in both human samples and mouse models of bronchopulmonary dysplasia (Rogers et al., 2015; Robbins et al., 2016). The epigenetic mechanisms for the regulation of the miR-17–92 cluster expression in cancer, and in NHL specifically, are not well explored and further investigation is therefore required to identify the role of miR-92a in malignancy and specifically in lymphomagenesis. In NHL patient whole blood gDNA, statistically significant increased levels of mean methylation were observed in region 1 of the *MIR17HG* CGI when compared to healthy controls. Although the mean difference between the two cohorts was not substantial, several individuals in the NHL patient cohort presented high levels of genomic DNA methylation across these targeted regions. Aberrant methylation of levels were observed in individuals diagnosed with specific subtypes of NHL when compared to controls, BL being the most prominent. An aggressive subtype of NHL, the BL cell line Raji was identified to exhibit increased methylation in these same regions in the cell line populations, and previous studies have reported a similar increase in methylation levels to those identified in this study in other miRNA promoter regions, in both BL cell lines and BL tumours (Mazzoccoli et al., 2018; Mazzoccoli et al., 2019). Increased methylation was also observed between CLL individuals and controls in region 1 of the *MIR17HG*, and a previous study identified that aberrant methylation of miRNA promoter regions in circulating B-cells of CLL patients was associated with abnormal miRNA expression when compared to healthy B-cells. Increased expression of miR-92a has been implicated in CLL malignancy, specifically in lymph node proliferation centres (Szurián et al., 2017). As the case-control cohort in this study is comprised of whole blood gDNA rather than specifically PBMC or B-cell DNA these previous results cannot be replicated, but the similar rates of increased methylation of DNA can be considered, and further investigation into miRNA promoter methylation in CLL is required. It should be considered that, although differential methylation was observed between subtypes in this study, DNA hypermethylation has long been regarded as a hallmark of cancer, and the aberrant methylation observed in these regions may be a result of genome-wide increases in DNA methylation rather than a consequence of dysregulation of specific genes or promoter regions, endorsing further investigation into the role of DNA methylation in these specific NHL subtypes.

The findings of this study support previous conclusions regarding the significance of miR-92a in B-cell malignancy and reinforce the importance of investigating its specific role in NHL. Several previous studies have identified miR-92a as being implicated in dysregulation and suppression of tumour suppressors, including PTEN (Xiao et al., 2008) and PHLPP2 (Rao et al., 2012) in MCL models and VHL in CLL B-cells (Ghosh et al., 2009). This same may be true of TET2, wherein our studies identified a trend of dysregulation in some cells, along with additional novel findings which warrant further investigation. Differential expression of *TET2* was seen across NHL cell lines and malignant tissues compared to controls, with specifically increased expression of *TET2* in Mino, Raji, and Toledo cell lines alongside DLBCL and FL tumour tissue compared to controls. However, when taking into account the increased miR-92a-5p expression in the cell lines, NHL tumour tissue, and increased miR-92a-3p expression in the Mino cell line compared to controls, it is suggestive that miR-92a-5p and −3p may not be strong negative regulators of *TET2* within these subtypes. Further assessment of the functional role of miR-92a on *TET2* expression is required, feasibly with the utilisation of miRNA mimics and inhibitors, to determine the effect of miR-92a-5p and −3p expression and inhibition in NHL cell models.

SU-DHL-4 and Raji cell lines showed significant hypermethylation in the studied CpGs within the *TET2* upstream promotor region when compared to controls and to Mino and Raji cell lines. This novel finding correlated with expected downregulation in *TET2* expression in the SU-DHL-4 cells, along with a similar trend observed in the Raji cells compared to Mino and Toledo cell lines in previous studies (Chiba, 2016; Chiba, 2017). Although *TET2* mRNA generally reported increased expression in cell lines and tumours, with the exception of SU-DHL-4, when compared to controls in our findings, *TET2* methylation in our target region negatively correlated with expression in the NHL cell lines, indicating that the CpGs assayed in this region may be involved in the regulation of *TET2* transcription and expression. *TET2* overexpression in NHL is not well explored when compared to well-documented trends of downregulation in *TET2* within various haematological malignancies (Chiba, 2017); however, previous studies have established this same trend of *TET2* over-expression in CLL as observed in our study (Hernández-Sánchez et al., 2014). A possible explanation for this finding is *TET2* overexpression in cells of some aggressive and indolent NHL subtypes is an innate immune response, involving tumour suppressor and DNA repair pathways, to the specific subtype of malignancy. Examination of TET2 protein and of downstream pathways of TET2 related expression regulation may assist in determining the translational consequence of *TET2* overexpression in these NHL samples and cell lines.

Previous studies have identified *TET2* variants in DLBCL as being associated with malignancy via the hypermethylation of pro-tumorigenic genes (Liu et al., 2017). Genotyping of the *TET2* gene in the specific NHL cell models and tumours samples may therefore provide greater understanding into the potential presence of disease-causing *TET2* mutations, which may contribute to disease phenotypes; as highlighted by differential expression DLBCL cell lines SU-DHL-4 and Toledo, both of which are DLBCL cell models. DNA methylation studies of CpGs in the *TET2* upstream promotor region across both NHL tumour-derived and control PBMC samples showed consistently low levels of methylation, and a similar trend was observed in the methylation of genomic DNA in the NHL case-control cohort. Differential methylation was observed in the *TET2* regulatory region between the case and control cohort; however, this difference may again not be biologically significant (Gruber et al., 2002; Tost and Gut, 2007). It is therefore unlikely that localised, as well as genome-wide, aberration of DNA methylation in CpGs in this region are directly involved in TET2 expression regulation in these NHL cohorts.

Clinical data from specific DLBCL tumour samples presented interesting characteristics and relationships when both *TET2* and miR-92a expression were considered (Table 1). One tumour sample displayed Reed-Sternberg-like cells during the histological examination, while our studies showed significantly decreased *TET2* expression compared to other DLBCL and FL tumour samples, as well as compared controls considered (Supplementary Figure S1E). Hypermethylation within our studied CpGs was not noted, therefore a likely cause of decreased *TET2* expression in this outlier is the presence of a functional mutation in *TET2*. Reed-Sternberg-like cells have been previously associated with TET2 mutations (Venanzi et al., 2021) along with diminished 5 hmC levels (Siref et al., 2020). Genotyping the tumour samples would assist in determining whether a polymorphism or variant in *TET2* is the cause of downregulated *TET2* levels, thereby further supporting previous findings regarding *TET2* as a potential marker and driver of malignancy in NHL.

## Concluding Remarks

The role of *TET2* in NHL has been previously documented; however, the mechanisms driving *TET2* expression regulation, such as DNA methylation, are not well explored. We identified in this study that aberrant methylation of several CpGs in the regulatory region of *TET2* correlated with *TET2* mRNA expression in NHL cell models. These findings are novel and may indicate that methylation of these regions may play a functional role in *TET2* expression. This same relationship was not observed in NHL tumour samples or in NHL patient genomic DNA. Although a target of miR-92a, and aberrant miR-92a-3p and miR-92a-5p expression was observed in NHL cell models and NHL tumours, an association between miR-92a and *TET2* was not observed in this study. Further assessment of the functional role of miR-92a-*TET2* in human cell models is required; as is, ultimately, assessment in clinical cohorts. Although *TET2* may not be comprehensively regulated by miR-92a, these novel findings regarding dysregulated methylation of *TET2* regulatory regions in NHL is an intriguing relationship warranting further investigation and reinforces the prominence of *TET2* as an element in NHL pathogenesis and malignancy.

## Data Availability

The raw data supporting the conclusions of this article will be made available by the authors, without undue reservation to any qualified researcher.
