# Supplementary material for: Epigenetic Regulation of miR-92a and TET2 and Their Association in Non-Hodgkin Lymphoma
Source: Front Genet. 2021 Nov 26;12:768913. doi: 10.3389/fgene.2021.768913 (PMC8661906; doi:10.3389/fgene.2021.768913)
Supplement: Supplementary file 1 [file DataSheet1.docx]

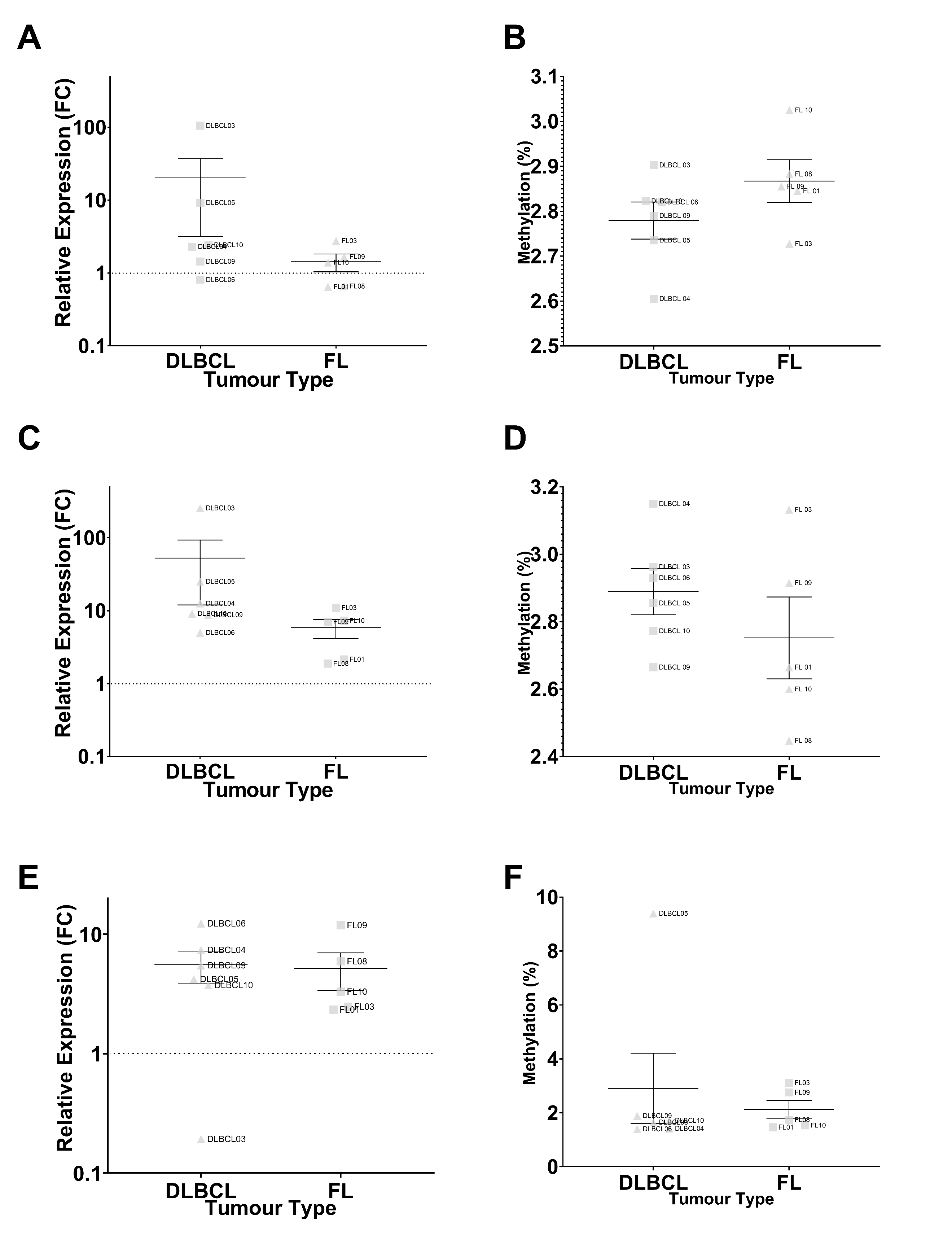


**Supplementary Figure 1: Expression miRNA 92a-3p, miRNA 92a-3p, and TET2 mRNA, and promoter DNA methylation of CpGs in two regions of the promoter CGI of the miR-17~92 cluster and one region in the promoter CGI of the TET2 gene in NHL tumours, with individual tumour samples marked and annotated. (A)** miR-92a-3p expression **(B)** *MIR17HG* region 1 methylation **(C)** miR-92a-5p expression **(D)** *MIR17HG* region 2 methylation **(E)** *TET2* expression **(F)** *TET2* methylation.
